# Clinical Characteristics and Influencing Factors of Feeding Intolerance After Surgery for Neonatal Necrotizing Enterocolitis

**DOI:** 10.3390/children12020127

**Published:** 2025-01-24

**Authors:** Mengting Hu, Fan Wu, Zhikai Fu, Yasi Zhang, Xinmin Ju, Zheng Chen, Xiaolu Ma, Yuanyuan Zhang, Wei Shi

**Affiliations:** 1 Neonatal Intensive Care Unit, Children’s Hospital, Zhejiang University School of Medicine, Hangzhou 310052, China; mengthu@zju.edu.cn (M.H.); 3220100404@zju.edu.cn (F.W.); 3220104223@zju.edu.cn (Z.F.); 3220105067@zju.edu.cn (Y.Z.); 22418780@zju.edu.cn (X.J.); chenz@zju.edu.cn (Z.C.); 6198007@zju.edu.cn (X.M.); 2 National Clinical Research Center for Child Health, National Children’s Regional Medical Center, Hangzhou 310052, China; chzyy@zju.edu.cn; 3 Department of Pulmonology, Children’s Hospital, Zhejiang University School of Medicine, Hangzhou 310052, China

**Keywords:** necrotizing enterocolitis, surgery, feeding intolerance, influencing factors

## Abstract

Background: Feeding intolerance (FI) following surgery for neonatal necrotizing enterocolitis (NEC) can impact recovery and prognosis, making the early identification of FI risk essential for optimizing management and improving outcomes. Methods: We retrospectively collected data from patients who underwent surgery for NEC between January 2013 and December 2023. Multivariate binary logistic regression was performed to identify independent factors influencing postoperative feeding intolerance. Results: Of the 519 infants enrolled in this retrospective study, 155 (29.9%) were diagnosed with feeding intolerance, while 364 (70.1%) were identified as having feeding tolerance. Compared to infants with feeding tolerance, those with feeding intolerance had lower birth weight, smaller gestational age, and lower Apgar scores (all *p* < 0.01). A 5 min Apgar < 7 (OR 4.794; 95%CI 1.339–17.156), the interval between diagnosis and surgery (OR 0.973; 95%CI 0.947–1.000), and primary anastomosis resection (OR 0.278, 95%CI 0.139–0.555) were identified as significant factors influencing postoperative feeding intolerance. The results remained consistent after performing propensity score matching analysis. Feeding intolerance may result in prolonged hospital stays, and more complications such as retinopathy of prematurity, intestinal failure-associated liver disease, and intraventricular hemorrhage. Conclusions: A lower 5 min Apgar score, shorter interval from diagnosis to surgery and intestine resection with ostomy are associated with a higher incidence of FI after surgery. FI after NEC surgery can prolong recovery and increase family burden.

## 1. Introduction

Necrotizing enterocolitis (NEC) is a life-threatening gastrointestinal disease characterized by intestinal inflammation and necrosis [1]. It predominantly affects premature infants, with an incidence of approximately 7% in very low birth weight (VLBW) infants [2]. The primary treatments for NEC include antibiotics, gastrointestinal decompression, and surgery. Around 20–40% of NEC cases require surgical intervention [3], with the mortality rate rising to 30–35% in these patients [4], and reaching as high as 50.9% in VLBW infants [5]. Effective perioperative management is crucial for promoting recovery and improving postoperative outcomes [6].

Postoperative complications in NEC patients may include intestinal stricture [7]^,^ short bowel syndrome [8], postoperative infections, and intestinal failure. Feeding intolerance (FI) is another common postoperative complication, presenting with symptoms such as abdominal distension, vomiting, and diarrhea [9]. These symptoms are typically associated with the recovery of intestinal structure and function, influenced by factors such as intestinal motility, barrier integrity, and inflammatory responses [10]. Postoperative feeding intolerance contributes to prolonged hospital stays and an increased risk of gastrointestinal complications. Moreover, sustained malnutrition can have long-term consequences on neurodevelopment, potentially impairing growth, attention, behavior, and social and emotional development [11].

With the growing adoption of the Enhanced Recovery After Surgery (ERAS) approach, there is increasing focus on postoperative management in children [12]. However, research on FI following surgery for NEC remains scarce. To address this gap, we aim to conduct a retrospective study to identify the influencing factors for FI after NEC surgery. The aim of this study is to offer guidance for optimizing postoperative care and enhancing the prognosis of children.

## 2. Methods

### 2.1. Study Design, Setting, and Participant Selection

This retrospective study was conducted in Zhejiang University Children’s Hospital, a leading tertiary perinatal care center. The study population comprised patients diagnosed with NEC at the hospital between January 2013 and December 2023. Inclusion criteria: neonates with a confirmed diagnosis of NEC, as retrieved from the electronic medical record system and consistent with the modified Bell staging system [13]. Surgical indications included NEC unresponsive to medical management or NEC complicated by intestinal perforation [14]. Exclusion criteria: (1) intestinal malformations; (2) cyanotic congenital heart disease; (3) inherited metabolic disorders; (4) spontaneous intestinal perforation; (5) discharged against medical advice; (6) incomplete data; and (7) death. A total of 908 cases were initially collected. Of these, 290 were excluded due to the presence of conditions listed in the exclusion criteria or the absence of surgical intervention. An additional 43 cases were excluded due to the withdrawal of medical care. A total of 56 cases resulted in death, including 15 attributed to shock or multiple organ failure without surgery and 41 occurring after surgery. Among the 41 postoperative deaths, 18 were caused by severe intestinal necrosis, while 23 were due to serious postoperative complications. Ultimately, 519 patients remained in the study. Based on our experience, the policy for postoperative nutritional management is as follows: (1) The initiation of enteral feeding after NEC surgery varies from three days to several weeks, depending on the severity of NEC, as determined by the surgical team. (2) Human breast milk (from mothers or donors) is the preferred feed. (3) Semi-elemental or amino acid–based formula may be considered if human breast milk is unavailable. (4) The volume of feeds should be gradually advanced at a rate of 15–24 mL/kg/d [15,16]. (5) Parenteral nutrition is administered prior to achieving adequate enteral feeding. This study was conducted in accordance with the principles outlined in the Declaration of Helsinki and was approved by Zhejiang University Children’s Hospital Institutional Review Board (2024-IRB-0356, approved on 11 December 2024).

### 2.2. Definitions

(1) Feeding intolerance (FI): this is defined as the interruption of feeding due to the following conditions: abdominal distension, color changes, visible bowel loops or pain, abnormal gastric residuals (gastric residuals greater than 100% of the previous feed, or the presence of bile, blood, or stool-like substances), vomiting and/or reflux, abnormal stools (such as mucus or blood), and related cardiopulmonary events [17].
(2) Small for gestational age infant (SGA): this is defined as birth weight <  10th percentile according to the Fetal Medicine Foundation (FMF) fetal and neonatal population weight charts [18].
(3) Medically treated patent ductus arteriosus (PDA): this is defined as the use of indomethacin, ibuprofen, or acetaminophen to promote the functional closure of the ductus arteriosus and improve hemodynamic stability in the presence of hemodynamically significant PDA (hsPDA) [19,20].
(4) Bronchopulmonary dysplasia (BPD): according to the NIH 2018 diagnostic criteria for BPD, preterm infants born at <32 weeks of gestation who require supplemental oxygen (FiO_2_ > 21%) for at least three consecutive days within the first 28 days after birth, and continue to need oxygen or respiratory support at 34 weeks of corrected gestational age, can be diagnosed with BPD [21].
(5) Retinopathy of prematurity (ROP): this is based on International Classification of Retinopathy of Prematurity, Third Edition [22].
(6) Short bowel syndrome (SBS): this is defined as patients requiring parenteral nutrition for more than 42 days due to the extensive resection of the intestine or residual small bowel length of less than 25% predicted by gestational age [23].
(7) Extrauterine growth restriction (EUGR): weight at discharge less than the 10th centile (cross-sectional definition) [24].
(8) Intraventricular hemorrhage (IVH): this is based on the ultrasound and evaluated according to the original classification system proposed by Papile et al., which categorizes the hemorrhage into four grades based on the location and extent of the bleeding [25].
(9) Sepsis and septic shock: this is based on the Phoenix sepsis criteria [26].
(10) Surgical strategies are as follows: (1) primary anastomosis resection: limited NEC disease, with stable internal environment; (2) intestine resection and ostomy: when faced with multi-focal disease; and (3) primary peritoneal drainage: unstable to undergo laparotomy, with the goal of stabilizing the patient until laparotomy can be performed [27].

### 2.3. Data Collection

Clinical data were retrospectively collected from the hospital’s electronic medical records, including demographic information (e.g., birth weight, gestational age, and Apgar scores), postoperative data (e.g., the type of surgery, postoperative complications, and nutritional support), and outcome data (e.g., the length of hospital stay, complication occurrence, long-term growth, and development outcomes). All data were verified by the research team for accuracy.

### 2.4. Statistical Analysis

The study population was stratified into two groups: the feeding intolerance (FI) group and the feeding tolerance (FT) group. Normality tests were conducted for continuous variables. For normally distributed data, values were expressed as mean ± standard deviation and compared using *t*-tests. For non-normally distributed data, values were presented as median (inter quartile range) and compared using Mann–Whitney tests. Categorical variables were expressed as frequency (percentage) and compared using chi-square tests. Univariate and multivariate binary logistic regression analyses were used to identify risk factors. Further propensity score matching analysis was conducted with a caliper value set at 0.02, balancing variables such as birth weight and gestational age. All statistical analyses were conducted using SPSS version 26.0, with a two-sided significance level set at 0.05.

## 3. Results

### 3.1. Demographic Characteristics

A total of 908 cases were initially collected. Based on the exclusion criteria, 389 patients were excluded, and ultimately, 519 patients were included in the study, with 155 in the FI group and 364 in the FT group (Table 1). The baseline characteristics of both groups are presented in Table 1. In terms of maternal factors, there were no significant differences between the two groups, such as gestational hypertension, gestational diabetes, umbilical abnormality, antenatal corticosteroids, multiple pregnancy (*p* > 0.05). In terms of neonatal characteristics, no significant differences were found between the two groups regarding sex, cesarean section rate, or the proportion of SGA. But infants in the FI group had smaller gestational age (30.4 (28.4, 33.0) vs. 32.9 (30.3, 35.6) days, *p* < 0.001), lower birth weight (1450 (1179, 1760) vs. 1780 (1400, 2400) g, *p* < 0.001) and lower 5 min Apgar score (10.0% vs. 1.5%, *p* < 0.001) compared to those in the FT group. We also compared the conditions of both groups during the first week after birth and found that infants in the FI group required a longer duration of mechanical ventilation (4 (0, 7) vs.1 (0, 6) days, *p* < 0.001) and had greater antibiotic exposure (5 (3, 7) vs. 4 (0, 7) days, *p* < 0.001) than those in the FT group.

### 3.2. Postoperative Characteristics

We compared the postoperative conditions between the two groups of NEC patients and found that infants in the FI group had a shorter interval between diagnosis and surgery (2 (1, 5) vs. 3 (1, 21) days, *p* = 0.002) compared to those in the FT group. Regarding surgical methods, a higher proportion of patients in the FI group underwent intestine resection and ostomy (78.7% vs. 45.6%, *p* < 0.001), while fewer patients in the FI group underwent primary anastomosis resection (14.2% vs. 45.6%, *p* < 0.001). Compared to the FT group, the length of the residual bowel in the FI group was shorter (79 (65, 100) vs. 90 (72, 115), *p* = 0.004). Additionally, a correlation analysis was conducted between the length of the residual bowel and the time to achieve enteral autonomy, revealing a linear relationship with an R value of −0.436 (*p* < 0.001) (Table S1). Additionally, the time to initiation of enteral feeding was longer in the FI group compared to the FT group (88.4%vs.65.1%, *p* = 0.001) (Table 2).

### 3.3. Analysis of Influencing Factors Associated with Feeding Intolerance

Multivariate binary logistic regression analysis was performed on the variables that showed significance in the univariate analysis. The following factors were ultimately identified as influencing factors for the occurrence of FI: 5 min Apgar < 7 (OR 4.794; 95%CI 1.339–17.156), interval between diagnosis and surgery (OR 0.93; 95%CI 0.947–1.000), and primary anastomosis resection (OR 0.278; 95%CI 0.139–0.555) (Table 3).

### 3.4. Sensitivity Analysis

We performed PSM using birth weight, gestational age, sex and SGA as balanced variables, followed by binary logistic regression analysis. The results remained consistent with the primary analysis when comparing infants in the FI and FT groups. Additionally, we identified postoperative fasting time > 5 days (OR 2.642; 95%CI 1.231–5.669) as an independent influencing factor for postoperative feeding intolerance (Table 2 and Table S3).

### 3.5. Prognosis

We found that infants in the FI group had significantly longer durations of mechanical ventilation, overall oxygen therapy, antibiotic use, and parenteral nutrition during their course of illness (*p* < 0.05, Table 4). In the FI group, patients who underwent colostomy experienced a delayed stoma closure. A higher proportion of infants in the FI group had comorbidities such as ROP, septic shock, intestinal failure-associated liver disease (IFALD) and IVH (*p* < 0.05).

## 4. Discussion

In this retrospective study, we collected and analyzed the clinical data of 519 patients after surgery for NEC. We identified 5 min Apgar score < 7, the interval between diagnosis and surgery, and the type of surgical procedure (primary anastomosis resection) as significant factors influencing postoperative feeding intolerance.

The mechanism of feeding intolerance after NEC is unclear, and may be due to reduced gastrointestinal peristalsis [28], systemic inflammatory response, and intestinal flora imbalance [29]. In our study, we identified that early postoperative physiological conditions, such as lower birth weight, gestational age, and lower Apgar scores, were significantly associated with an increased risk of feeding intolerance. Lower birth weight and gestational age typically indicate an immature physiological state in neonates, with an underdeveloped digestive system that renders them more susceptible to FI after surgery [30,31]. Additionally, poor Apgar scores and the need for neonatal resuscitation reflect neonatal distress at birth, which is often associated with multi-organ dysfunction and a compromised immune response [32], thereby exacerbating postoperative complications [33]. Our study identified that the duration of mechanical ventilation and antibiotic exposure within one week after birth were significantly longer in the FI group. A multicenter study [34] found that lower birth weight, prolonged mechanical ventilation time, and an Apgar score ≤ 7 at 5 min were associated with extended empirical antibiotic treatment, which may increase the risk of FI. Another study [35] analyzed the gut microbial diversity and composition in infants with FI and confirmed the association between intestinal microecological imbalances and the development of FI. Antibiotics have a significant influence on the intestinal microbiome. The ecological consequences of antibiotic use may be more pronounced and persistent when administered during the first weeks of life [36].

Pneumoperitoneum remains the only absolute indication for surgery in patients with NEC, while physiological deterioration despite medical treatment serves as a relative indication for surgical intervention [37]. Determining the optimal timing for surgical intervention in NEC patients remains challenging. We found that patients in the FI group had a shorter interval between diagnosis and surgery, which was identified as an independent risk factor for the development of FI. This indicates that patients in the FI group experience more rapid disease progression, which significantly impacts postoperative feeding outcomes.

Compared to primary anastomosis resection, resection with stoma formation was more commonly associated with feeding intolerance. However, infants with better overall intestinal health may have undergone primary anastomosis. Conversely, infants with severe intestinal lesions or a poor overall condition may require an ostomy during the initial surgery. Thus, the type of surgery may serve as a marker of intestinal health and, consequently, an indicator of the overall risk of FI. A prospective randomized controlled trial demonstrated superior outcomes for primary anastomosis resection in infants with necrosis and/or perforation caused by necrotizing enterocolitis. The study demonstrated that infants who underwent primary anastomosis achieved full intestinal autonomy 21 days earlier than those who had a stoma [38]. Due to the lack of a powered randomized controlled trial, no definitive recommendation can be made regarding the choice of one operative strategy over another [39]. Further prospective studies with larger sample sizes are needed to provide more definitive conclusions.

Nutritional support is a key component of recovery in these infants [40]. Research has shown that an early initiation of enteral feeding after surgery can reduce hospital stay and lower the risk of complications [41,42]. Adequate nutrition helps maintain tissue integrity, supports immune function, and promotes growth and development, especially during periods of increased metabolic demands, such as post-surgery [43,44,45]. There is no guideline regarding the optimal timing for the initiation of postoperative enteral feeding. Based on our clinical experience, enteral nutrition should be reintroduced when bowel sounds are present, gastric decompression yields no bile, the abdomen is soft, and vomiting has ceased. Given that infants in the FI group had lower gestational age and birth weight compared to those in the FT group, delayed feeding may indicate impaired intestinal function, leading to a slower recovery in the FI group relative to the FT group. These confounding factors need to be further adjusted in subsequent studies to enhance the accuracy and reliability of the findings.

Our study also found that infants with feeding intolerance were more likely to experience adverse clinical outcomes, including prolonged mechanical ventilation, extended oxygen therapy, longer antibiotic use, and increased reliance on intravenous nutrition. These factors reflect the multiple challenges feeding intolerance patients face during their recovery, which not only increases the treatment burden but also potentially impacts long-term prognosis. Moreover, a higher proportion of infants in the feeding intolerance group had comorbidities such as ROP, septic shock, IFALD, and IVH, which further highlights the significant impact of feeding intolerance on neonatal outcomes. Alternatively, preterm infants may be at a higher risk for comorbidities such as ROP, BPD, and IVH. Since we did not compare preterm infants with full-term infants who had FI, it is important to acknowledge that the underlying prematurity-related conditions could confound our findings. We controlled factors such as birth weight, gestational age, and whether the infant was SGA through propensity score matching (PSM) to minimize bias. Further studies with larger sample sizes are required to confirm these findings and better assess the relationship between prematurity and postoperative FI.

Several limitations should be addressed in future research to further optimize and improve the findings. Firstly, this study is a retrospective, single-center analysis with a relatively small sample size, which limits the generalizability and external validity of the results. Secondly, although several potential influencing factors were identified, some factors that may influence feeding intolerance were not included in the analysis, particularly variations in feed practice such as the type and rate of feed.

## 5. Conclusions

This study describes the clinical characteristics of FI following surgery for NEC and identifies several key factors influencing postoperative feeding intolerance. These findings provide scientific evidence supporting the early identification of feeding intolerance in neonates with NEC after surgery. However, influencing factors need to be further validated in larger sample sizes and multicenter studies to ensure their broader applicability and reliability in clinical practice.

## Figures and Tables

**Table 1:** The comparison of maternal and neonatal characteristics between feeding intolerance and feeding tolerance groups.

Characteristics	Infants, No. (%) (N = 519)		*p* Value
	Feeding Intolerance (*n* = 155)	Feeding Tolerance (*n* = 364)	
Maternal characteristics			
Gestational hypertension	19 (12.3%)	37 (10.2%)	0.482
Gestational diabetes	13 (8.4%)	31 (8.5%)	0.994
Umbilical abnormality	11 (7.1%)	25 (6.9%)	0.925
Placental abnormality	11 (7.1%)	19 (5.2%)	0.396
Amniotic fluid abnormality	6 (3.9%)	11 (3.0%)	0.623
Antenatal corticosteroids	12 (7.7%)	16 (4.4%)	0.124
Multiple pregnancy	60 (38.7%)	115 (32.5%)	0.117
Neonatal characteristics			
Male sex	92 (59.4%)	197 (54.1%)	0.272
Gestational age, median (IQR), wk	30.4 (28.4, 33.0)	32.9 (30.3, 35.6)	<0.001
Birth weight, median (IQR), g	1450 (1179, 1760)	1780 (1400, 2400)	<0.001
SGA	24 (15.5%)	42 (11.5%)	0.221
Cesarean delivery	98 (63.2%)	247 (67.9%)	0.287
5 min Apgar < 7	13 (10.0%)	4 (1.5%)	<0.001
PDA medication	16/60 (26.7%)	20/73 (27.4%)	0.928
PDA ligation	5/60 (8.3%)	9/73 (12.3%)	0.455
Surfactant use	59 (38.1%)	77 (21.2%)	<0.001
Intubation in delivery room	9 (5.8%)	22 (6.0%)	0.917
One week after birth			
Days of mechanical ventilation, median (IQR), d	4 (0, 7)	1 (0, 6)	<0.001
Days of antibiotic exposure, median (IQR), d	5 (3, 7)	4 (0, 7)	<0.001
Sepsis	67 (43.2%)	161 (44.2%)	0.833
Age of onset, median (IQR), d	14 (9, 23)	13 (7, 22)	0.182

wk: week; d: day; SGA: small for gestational age infant; PDA: patent ductus arteriosus.

**Table 2:** The comparison of postoperative characteristics between feeding intolerance and feeding tolerance groups.

Characteristics	Infants, No. (%) (N = 519)		*p* Value
	Feeding Intolerance (*n* = 155)	Feeding Tolerance (*n* = 364)	
Interval days between diagnosis and surgery, median (IQR), d	2 (1, 5)	3 (1, 21)	0.002
Surgical approach			
Primary anastomosis resection	22 (14.2%)	166 (45.6%)	0.000
Intestine resection and ostomy	122 (78.7%)	166 (45.6%)	0.000
Non-intestine resection and ostomy	7 (4.5%)	28 (7.7%)	0.187
Primary peritoneal drainage	4 (2.6%)	4 (1.1%)	0.210
Length of residual bowel, median (IQR), cm	79 (65, 100)	90 (72, 115)	0.004
Necrosis area			
Small intestine	84/120 (70.0%)	98/171 (57.3%)	0.036
Both large intestine and small intestine	29/120 (24.2%)	53/171 (31.0%)	0.197
Postoperative fasting time > 5 days	137 (88.4%)	273 (65.1%)	0.001

d: day.

**Table 3:** Identification of independent influencing factors for postoperative feeding intolerance in NEC by multivariable binary logistics regression.

Variables *	B	OR	95% CI	*p* Value
5 min Apgar < 7	1.567	4.794	1.339–17.156	0.016
Interval days between diagnosis and surgery	−0.027	0.973	0.947–1.000	0.048
Primary anastomosis resection	−1.281	0.278	0.139–0.555	0.000

* Gestational age, birth weight, 5 min Apgar < 7, days of ventilator use within the first week of life, days of antibiotic use within the first week of life, surfactant use, interval days between diagnosis and surgery, bowel resection and anastomosis, bowel resection and fistula formation, and postoperative fasting > 5 days were included in the multivariate binary logistic regression analysis.

**Table 4:** The comparison of prognosis between feeding intolerant and feeding tolerant groups.

Prognosis	Infants, No. (%) (N = 519)		*p* Value
	Feeding Intolerance (*n* = 155)	Feeding Tolerance (*n* = 364)	
Days of ventilation support, median (IQR), d	2 (1, 5)	1 (1, 2)	<0.001
Days of oxygen supplementation, median (IQR), d	10 (1, 36)	1 (1, 5)	<0.001
Days of antibiotic treatment, median (IQR), d	28 (19, 40)	20 (15, 31)	<0.001
Duration of parenteral nutrition, median (IQR), d	38 (25, 60)	13 (8, 22)	<0.001
Stoma closure age, median (IQR), d	129 (94, 185)	106 (85, 140)	0.005
BPD	23 (14.8%)	34 (9.3%)	0.070
ROP	35 (22.6%)	25 (6.9%)	<0.001
Septic shock	20 (12.9%)	23 (6.3%)	0.013
PVL	2 (1.3%)	5 (1.4%)	0.941
IVH	35 (22.6%)	43 (11.8%)	0.002
IFALD	49 (31.6%)	45 (12.4%)	<0.001
SBS	10 (6.5%)	8 (2.2%)	0.016
Discharge situation			
Age, median (IQR), d	77 (57, 105)	52.5 (40, 75)	<0.001
Weight, median (IQR), g	3285 (2680, 3530)	3030 (2580, 3500)	0.181
Length, median (IQR), cm	49.5 (47, 52)	49 (46, 50)	0.035
Head circumference, median (IQR), cm	34.5 (33.5, 36.0)	34 (32.5, 35.5)	0.064
EUGR	31 (20.0%)	56 (15.4%)	0.634
Breast feeding	38 (24.5%)	95 (26.1%)	0.656
Amino acid-based formula	74 (47.7%)	117 (32.1%)	0.001

d: day; BPD: bronchopulmonary dysplasia; ROP; retinopathy of prematurity; PVL: periventricular leukomalacia; IVH: intraventricular hemorrhage; IFALD: intestinal failure-associated liver disease; SBS: short bowel syndrome; EUGR: extrauterine growth restriction.

## Data Availability

The data supporting the findings of this study are available from the corresponding author upon reasonable request. The data are not publicly available due to privacy.

## Supplementary Materials

The following supporting information can be downloaded at: https://www.mdpi.com/article/10.3390/children12020127/s1, Table S1: The Spearman’s Rank Correlation between bowel length and days to achieve enteral autonomy; Table S2: The comparison of characteristics between feeding intolerance and feeding tolerance groups after PSM; Table S3: Identification of independent influencing factors for post-operative feeding intolerance in NEC by multivariable binary logistics regression after PSM.
