# Supplementary material for: Clinical Characteristics and Influencing Factors of Feeding Intolerance After Surgery for Neonatal Necrotizing Enterocolitis
Source: Children (Basel). 2025 Jan 24;12(2):127. doi: 10.3390/children12020127 (PMC11854438; doi:10.3390/children12020127)
Supplement: Supplementary file 1 [file children-12-00127-s001.zip › children-3423520-supplementary.pdf]

Table S1 The Spearman's Rank Correlation between bowel length and days to achieve enteral autonomy.

Variables	Variables	R	*P* value
Preserved bowel length	days to achieve enteral autonomy	-0.436	P<0.001

Table S2 The comparison of characteristics between feeding intolerance and feeding tolerance groups after PSM.

**Characteristics**	***Infants, No. (%) (N=294)**		***P* value**
	**Feeding intolerance (*n*=147)**	**Feeding tolerance (*n*=147)**	
Gestational age, median (IQR), wk	30.57(29.00-33.00)	30.86(28.86-33.00)	0.977
Birth weight, median (IQR), g	1470(1200-1770)	1500(1220-1780)	0.720
5min Apgar<7	11(7.5%)	2(1.4%)	0.009
Surfactant use	55(37.4%)	48(32.7%)	0.392
One week after birth			
Days of mechanical ventilation , median (IQR), d	4(0-7)	2(0-7)	0.337
Days of antibiotic exposure, median (IQR), d	5(3-7)	5(2-7)	0.575
Interval days between diagnosis and surgery,median (IQR),d	2(1-5)	6(1-24)	P<0.001
Surgical Approach			
Bowel Resection and Anastomosis	21(14.3%)	68(46.3%)	P<0.001
Bowel resection and fistula formation	118(80.3%)	65(44.2%)	P<0.001
Small intestine necrosis	81/115(704%)	43/67(64.2%)	0.382
Postoperative fasting time>5 days	130(88.4%)	105(71.4%)	P<0.001

*Adjusted for gestational age,birth weight,sex,SGA. wk:week ;d:day.

Table S3 Identification of independent influencing factors for post-operative feeding intolerance in NEC by multivariable binary logistics regression after PSM.

**Variables***	**B**	**OR**	**95%CI**	***P* value**
5min Apgar<7	1.665	4.286	1.093-25.545	0.038
Interval day between diagnosis and surgery	-0.034	0.966	0.938-0.995	0.023
Bowel resection and anastomosis	-0.883	0.414	0.194-0.881	0.022
Post-operation fasting >5 days	0.971	2.642	1.231-5.669	0.013

*5-minute Apgar score <7, interval days between diagnosis and surgery, bowel resection and anastomosis and postoperation fasting >5 days were included in the multivariate binary logistic regression analysis.
